# Mid-frequency song and low-frequency calls of sei whales in the Falkland Islands

**DOI:** 10.1098/rsos.220738

**Published:** 2022-11-09

**Authors:** Salvatore Cerchio, Caroline R. Weir

**Affiliations:** ^1^ African Aquatic Conservation Fund, P.O. Box 366, Chilmark, MA 02535, USA; ^2^ Falklands Conservation, Jubilee Villas, Ross Road, Stanley FIQQ 1ZZ, Falkland Islands

**Keywords:** *Balaenoptera borealis*, passive acoustic monitoring, low-frequency calls, song display, singing behaviour

## Abstract

Although sei whales (*Balaenoptera borealis*) are distributed throughout the globe, their behaviour and vocal repertoire are poorly described. We used passive acoustic monitoring to describe the vocal behaviour of sei whales in the Falkland Islands, between December 2018 and April 2019. We isolated more than 2000 low-frequency calls for manual classification, of which 510 calls with high signal-to-noise ratio were quantitatively measured. Five categories of stereotyped call types in the 15–230 Hz range were described, some with multiple subcategories. These included some similar to previously described calls (e.g. downsweeps), but others that were novel in acoustic structure and frequency band. In the mid-frequency range, we documented a highly stereotyped, hierarchically structured and rhythmically repetitive song display. Songs were arranged in phrases with a structure composed of repetitive sub-phrases, and a diverse variety of sounds in the 1–5 kHz range. Singing commenced in late February, despite the presence of whales and calls since early December, and continued through April. These acoustic properties and behavioural characteristics indicate that this is likely a male breeding display similar to songs and singing of other balaenopterids. This is the first detailed description of a song display for sei whales, highlighting the importance of the Falkland Islands.

## 1. Introduction

The sei whale (*Balaenoptera borealis*) occurs from the tropics to the polar regions in all ocean basins, with a documented global distribution that is primarily concentrated in temperate mid-latitude (20 to 55°) regions [1]. Like other migratory baleen whales, sei whale populations are believed to undertake seasonal movements between summer feeding areas in the higher latitude parts of their range, and winter breeding grounds located in the subtropics. Although widely distributed on a global scale, their finer-scale occurrence, ecology and behaviour remain relatively poorly documented due to their oceanic habits and unpredictable occurrence in many geographical regions [1], which has limited the scope for targeted field research. Additionally, frequent confusion with other similarly sized baleen whales such as Bryde's whales (*Balaenoptera edeni* and *Balaenoptera brydei*) has occurred in both whaling statistics and modern sighting surveys, which has further hindered knowledge of their distribution and ecology [2].

The waters around the Falkland Islands in the southwest Atlantic have recently been well documented as an important habitat for Southern Hemisphere sei whales [3–6]. Sei whales are present in the Falklands seasonally during the austral summer and autumn from November to June, and the region is considered to comprise coastal feeding habitat, well-documented in Berkeley Sound, Falkland Sound and the west coast of the Falklands, where they feed on shoaling prey including lobster krill (*Munida gregaria*) and the amphipod *Themisto gaudichaudii* [3,6,7]. Suitable foraging habitat is predicted to occur throughout the archipelago [4]. Population abundance estimated during February and March 2018 on the west coast of the Falklands ranged from 707 (CI [566, 877]) to 916 (CI [606, 1384]) animals [6]. Therefore, Weir *et al*. [6] suggested that this region supports a globally important feeding aggregation for a population that was severely reduced by twentieth century whaling, and a species considered globally Endangered on the IUCN Red List [8].

Low-frequency (LF; less than 200 Hz) calls have been attributed to sei whales in several parts of the world [9–13]. Tonal downsweeps in the approximately 100–40 Hz range were first identified as a characteristic vocal behaviour attributed to sei whales in the central North Pacific (off Hawaii [9]) and in the western North Atlantic (off Cape Cod [10]), and subsequently described in other geographical regions, including off Chile [12] and the Azores [14,15]. Repetitive downsweeps (i.e. those that occur in patterns of doublets, triplets or more, with intervals of approx. 3.5 s) have been reported by several of those studies and have been used as a means to distinguish sei whale downsweeps from similar LF calls produced by other balaenopterids (e.g. [16]). Additional tonal LF calls have also been reported for sei whales including upsweeps (35–75 Hz range) and ‘upsweep-downsweep’ calls (50–90 Hz range) in the Southern Ocean [11], and lower frequency downsweeps (50–20 Hz range) in the North Pacific and North Atlantic [9,13], respectively. Some authors have also described mid-frequency (MF) vocalizations in the 1.5–3.5 kHz range for North Atlantic sei whales [17,18], and the 200–1000 Hz range for Southern Hemisphere sei whales [19]; however, MF vocalizations have not been reported again in the North Atlantic despite some extensive recording efforts [15,20]. The reported MF sounds were described as a ‘*burst of…metallic pulses*' by Thompson *et al*. [17] recorded between Nova Scotia and Newfoundland during August, as broadband ‘*frequency modulated sweeps*’ by Knowlton *et al*. [18] recorded on the Scotian shelf during August and September, and as ‘*broadband…growls or whooshes*' by McDonald *et al*. [19] recorded west of the Antarctic Peninsula during February. Knowlton *et al*. [18] described the sounds as being arranged in phrases, using terminology typically used to describe songs, and inferring hierarchal structure. McDonald *et al*. [19] suggested that Knowlton *et al*. [18] and Thompson *et al*. [17] may have recorded reproductive song, distinguishing it from the lower frequency vocalizations that they had recorded for the species. Tremblay *et al*. [13] also reported song-like sequences of LF vocalizations from North Atlantic sei whales in Massachusetts Bay during September and October, comprising a series of LF downsweeps including 82–34 Hz downsweep doublets combined with newly described 50–30 Hz triplet and singlet downsweeps. The individual groupings of downsweeps reported by Tremblay *et al*. [13] were clearly stereotyped and suggestive of baleen whale song; however, the sequences were organized into somewhat variable patterns and the degree of stereotypy, repetitive rhythm and seasonal occurrence requires further evaluation.

This paper reports on vocalizations of sei whales collected during a long-term deployment of archival bottom-mounted acoustic recorders in Berkeley Sound, Falkland Islands. The primary aim of the deployments was to document the spatio-temporal occurrence of sei whales within Berkeley Sound, particularly at times when visual surveys were not viable (e.g. at night, during winter and during prolonged periods of poor weather). A fundamental component of the work was to describe the vocal repertoire and vocal behaviour of sei whales in the region. Here, we describe in detail a variety of low-frequency call types, several of which are previously unreported, as well as a novel mid-frequency stereotyped and rhythmically repetitive song display for sei whales.

## 2. Methods

### 2.1. Recording gear specifications

The passive acoustic monitoring (PAM) gear used in this study were OceanInstruments^NZ^ SoundTrap ST500-STD recorders, tethered to INNOVASEA Ascent AR acoustic releases and floated with an 11" buoy (8.4 kg buoyancy) approximately 2 m above a 60 kg iron anchor chain mooring on the seafloor. The SoundTrap recorder specifications indicated a flat response from 20 Hz to 60 kHz (±3 dB) with a −9 dB roll-off at 10 Hz, and a 34 dB re 1 V µPa^−1^ noise floor. Calibration information provided by the manufacturer for each specific recorder indicated that hydrophone sensitivity plus system gain ranged from −175.4 to −174.4 dB re 1 V µPa^−1^ among the three units used in Berkeley Sound. All recorders sampled at 16 bits and 24 kHz for a 12 kHz analysis band and recorded continuously without interruption or duty cycle during any single deployment.

### 2.2. Berkeley sound PAM effort

**Figure 1.**
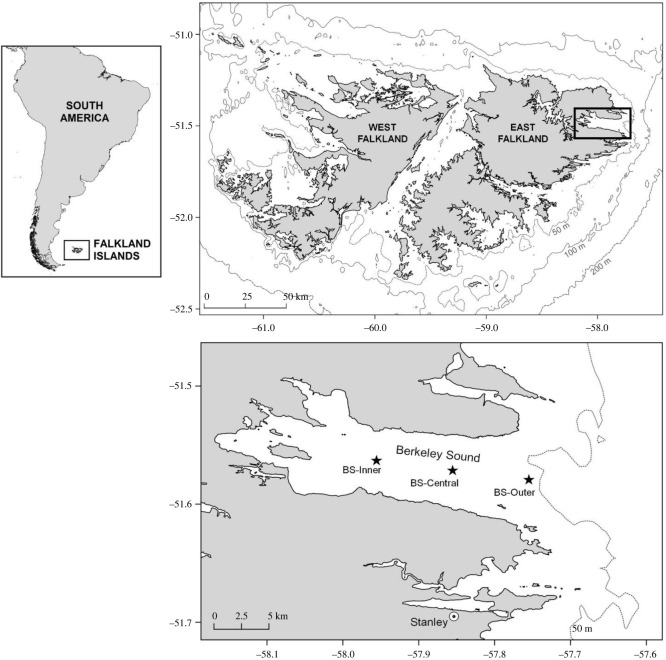
Location of study area, including positions of recorders during deployments in Berkeley Sound: BS-Outer, BS-Central and BS-Inner sites.

Three recorders were deployed in Berkeley Sound (BS), East Falkland, at approximately 7 km spacing (figure 1):

— Berkeley Sound Outer (BS-Outer): outermost site near the mouth of the Sound, approximately 5 km from the north and south coasts, depth: 45–47 m;
— Berkeley Sound Central (BS-Central): central site, approximately 3 km from the coasts, depth: 29 m; and
— Berkeley Sound Inner (BS-Inner): innermost site, approximately 3 km from the coasts, depth: 27–31 m.

The data presented here were collected primarily during a single deployment of the ST500 recorders between 5 December 2018 and 23 April 2019, and primarily from the BS-Central site (Deployment 1, amounting to 139 days or 3335 h of continuously recorded data), but supplemented with data from the BS-Outer site. The BS-Central recorder was chosen as the focal site for review due to its central location within the confines of the Sound, and best overlap between its detection range and coverage of concurrent boat-based surveys (see [21]). Additional recordings from BS-Central between 27 May 2019 and 30 September 2019 (Deployment 2) were also reviewed for the assessment of temporal distribution of sei whale vocalizations during the 2019 season. The gap of 34 days (23 April to 27 May 2019) between Deployments 1 and 2 was unintended and was the result of technical issues.

### 2.3. Acoustic data analysis

Most previously described sei whale vocalizations occur primarily in the LF range (less than 500 Hz). Consequently, all files were converted to a lower sample rate (SR) to facilitate the browsing and analysis of the data with smaller sized files. The software Avisoft-SASLab Pro was used to down-sample files, using the Sampling Frequency Conversion function. Since two studies have reported that sei whales may also emit vocalizations in the 1–3 kHz bandwidth [17,18], an SR of 11 025 Hz was chosen, providing an analysis bandwidth of 5512 Hz, so that MF vocalizations (500–5000 Hz) could also be detected if present.

In an effort to describe the vocalizations and vocal behaviour of sei whales in our sample, we have adhered to the following definitions. Any vocalization that does not occur in consistent rhythmically repetitive patterns is considered a ‘call’. Individual calls can be narrow-band tonal, frequency-modulated, amplitude-modulated or have complex spectral content. Call types can be stereotyped, with little variability between utterances, or have graded variation along a defined set of variability, and below we define several discrete call type categories, some with similar subcategories. Calls can be uttered as groupings in a series, or ‘motifs’ (short discrete patterns of a few calls), the presentation of which does not take on bouts of extended rhythmic repetition. Conversely, ‘song’ is defined by a vocal behaviour in which stereotyped patterns of vocalizations are repeated sequentially in a rhythmic manner for extended periods (several minutes to hours) without interruption. Singing behaviour following this description has been well described for other baleen whales (for example, humpback whales, *Megaptera novaeangliae* [22]; fin whales, *Balaenoptera physalus* [23,24]; blue whales, *Balaenoptera musculus* [25]; Omura's whales, *B. omurai* [26,27]). For those species, multiple singers are often heard simultaneously in a chorus, and all singers which have been sexed were male.

An initial assessment of vocalizations was conducted on the data from BS-Central. The deployment was first analysed with the Low-Frequency Detection and Classification System (LFDCS) developed by Baumgartner & Mussoline [28], employing a user-defined library that included calls from four baleen whale species from the North Atlantic (sei whales, fin whales, humpback whales and North Atlantic right whales *Eubalaena glacialis*, as described in [16,29]). LFDCS is a contour-tracing algorithm that has been successfully used for detecting North Atlantic sei whales in both archival and real-time detection scenarios [16,30]. LFDCS first applies a protocol to condition the spectrogram by reducing tonal background noise (e.g. ship noise) and removing transient broadband signals. A contour-tracing algorithm is then applied to detect putative tonal signals and estimate the frequency variation of the putative call over time. Attributes of the pitch track (e.g. start frequency, end frequency, duration, slope of frequency variation) are extracted, and candidate calls are classified to call type based on comparison with a user-defined call library using quadratic discriminant function analysis [28]. The similarity between any classified call to a call type in the library is estimated using the Mahalanobis-distance (MD) metric, with lower values indicating greater similarity. An MD of 3.0 or less has been used for detection and classification of the 82–34 Hz sei whale downsweeps that are included in the North Atlantic call library [16]. For application of the North Atlantic call library to the Falklands data, we used the default LFDCS parameter settings (described in [16]) and an MD of 5.0, as a relaxed criterion for the detection of potential sei whale downsweeps similar to those described in the North Atlantic.

Detections of vocalizations identified by the LFDCS were reviewed on sound spectrograms generated in Raven Pro 1.5 [31]. Using the LFDCS detections of downsweeps as an indication of periods of time when sei whales were present, spectrograms of the 4.5 month period from 5 December 2018 to 23 April 2019 were browsed in both the LF and MF range to identify and log vocalizations. The continuous stream of 11 kHz SR acoustic files was loaded into Raven for spectrographic browsing using a window size and discrete Fourier transform (DFT) size of 8192 samples, 50% overlap, and Hann window. Spectrograms were viewed at 250 Hz bandwidth for reviewing the detected LF vocalizations, and the full 5512 Hz bandwidth for further assessing the presence of MF vocalizations, initially displaying three spectrogram lines of 60 s each on each Raven page. It was assumed that any LF vocalizations found in association with doublet downsweeps were produced by sei whales. Additionally, visual observations from cetacean boat surveys carried out in Berkeley Sound over the Deployment 1 period were used to document the presence of sei whales and whether any other baleen whale species may have been present during the recording period.

In addition to frequency-modulated tonal downsweeps, a variety of sei whale call types were identified, categorized and logged. Most observed call types were categorized based primarily on the frequency contour of the fundamental frequency, and whether it was a constant non-modulated tone, frequency-modulated, direction of modulation, and whether it contained an inflection point defining a change in contour slope. This was consistent with previous studies which have defined ‘downsweep’, ‘upsweep’ and/or ‘upsweep-downsweep’ calls [9–13]. An effort was made to identify and classify discrete stereotyped vocalizations that were recorded repeatedly on multiple days throughout the extended recording period and readily recognized by eye on the spectrographic display. Call categories were defined in an iterative review process between the two authors, initiating with (1) a preliminary definition of several discrete categories by the lead author, which was followed by (2) an extensive review by the second author to log and further categorize several thousand examples of calls, which were then (3) exhaustively reviewed by the first author to refine categories and subcategories and filter the logged calls to a dataset of the highest quality examples for quantitative measurement. Finally, after call type categories were finalized, the robustness of the categories was assessed with an additional inter-observer classification analysis: a naive observer who has extensive experience in baleen whale bioacoustics, but was unfamiliar with the study or dataset, was provided with the dataset of high-quality examples, along with the exemplar spectrograms and call type descriptions (as presented in Results), and asked to classify each call to its category.

In order to define the highest quality calls for measurement (step 3 above) the logged vocalizations (from step 2) were first ordered by received level (using Raven's Peak Power measurement), in order to review the loudest signals from presumably the closest animals. Next, a subset of 50–100 of the highest signal-to-noise ratio (SNR) calls for each call type category, assessed based upon background noise and clarity of the signal in the spectrogram, were selected for quantitative measurement. Signal measurements were made by tightly boxing calls on Raven spectrograms using the original 24 kHz SR files (not down-sampled), a window size of 8192 samples, a DFT size of 16 384 samples, 90% overlap and a Hann window, with a resultant temporal resolution (time grid spacing) of 34 ms and frequency resolution (frequency grid spacing) of 1.46 Hz. The measurements included duration (call end time − call begin time as measured by the temporal extent of the selection box, s), high frequency (maximum of selection box, Hz), low frequency (minimum of selection box, Hz), slope (frequency range/duration, Hz s^−1^) and peak frequency (frequency of peak power, Hz). The SNR (in dB) was estimated using Raven's Inband Power function and the procedure recommended by the Center for Conservation Bioacoustics (https://ravensoundsoftware.com/knowledge-base/signal-to-noise-ratio-snr), comparing identical time/frequency-band selections of signal and background noise. A minimum SNR of 16 dB was chosen as the criterion for inclusion of individual calls in the sample for quantitative measurement. Constraining measured calls to recordings with a high SNR increased the likelihood of accurate time-frequency parameter measurement from the spectrogram.

After identification and description of Falkland Island sei whale calls, a new LFDCS call library was created specifically including Falklands' sei whale calls, drawing exemplar calls from the data collected in BS during the period of peak sei whale occurrence. This procedure was conducted for separate objectives that are not reported here but were described in Cerchio *et al*. [32], to define the spatio-temporal distribution of sei whales in BS. Therefore, the LFDCS analysis was not meant to classify the entire repertoire of sei whale vocalizations, but was restricted to calls that were perceived to best represent sei whale presence. Two different call type categories, downsweeps and L-calls (described in the Results section), were found to be both numerous and stereotyped and therefore were selected to use in the call library. Three different subcategories of downsweep calls were incorporated to further improve the specificity of the detector. The performance of the Falklands-specific call library was quantified, and was substantially more effective than the North Atlantic library alone for detecting sei whale calls (see [32] for details).

During the initial manual spectrographic browsing of BS-Central recordings in the MF range, a stereotyped and repetitive song vocalization was discovered that has not been previously reported for sei whales (see Results section). In order to describe the temporal distribution of sei whale singing, an assessment of hourly presence of song at BS-Central was conducted. Long-term spectrograms were manually reviewed in Raven for the entirety of Deployment 1 and the first 33 days of Deployment 2, from 5 December 2018 through 30 June 2019; this period covered the primary period of sei whale presence in Berkeley Sound during 2019 as revealed by the LFDCS analysis of call detections [32]. The down-sampled files at a sample rate of 11 kHz were used, and spectrograms generated with a long integration period to emphasize the presence of the repetitive vocalizations in a long duration spectrogram line (FFT of 32 768 pts, 0% overlap, Hann window, viewing three spectrogram lines of 60 min duration each per page). The presence of song was systematically assessed for every hour, logging the duration of time within the hour in which singing was present. An assessment of whether there was one or multiple singers was made, based upon the presence of overlapping song phrase patterns. And a subjective rating of signal quality was assigned to each hour on a scale of 1 to 3 (based upon visual impression of received level, SNR and the presence of masking background noise). Duration of singing ‘bouts’ were calculated by summing successive logged periods of continuous singing with an arbitrary gap of less than 10 min; this period was chosen after taking into consideration the typical spacing between song phrases and assessing the distribution of observed gaps to infer when animals had stopped singing. The temporal distribution across the recording period and diel variation of sei whale singing were compared to the hourly occurrence of sei whale LF calls (downsweeps and L-calls), as evaluated by LFDCS with the Falklands specific call library and reporting occurrence with a MD threshold of 3.0 (as used in [16]).

## 3. Results

### 3.1. Sei whale low-frequency calls

The initial review of spectrograms from BS-Central during Deployment 1 revealed the presence of several categories of LF vocalizations that were attributed to sei whales. Species attribution was determined by: (1) the presence of known sei whale calls (downsweep doublets) among bouts of varied vocalizations; (2) the confirmed presence of numerous sei whales (and no other baleen whale species) during boat surveys in BS over this period [21]; (3) the use of data relating to the peak period of sei whale occurrence in the Falklands (Feb/Mar [3,21]) for classification of call types; and (4) the focus on data from BS-Central, which should limit detected calls to whales within the confines of Berkeley Sound, and thus present the best comparison with the species identifications confirmed during boat surveys. During Deployment 1 of the recorders, there were 16 days of boat surveys conducted between 25 January and 23 April 2019, amounting to 114.2 h and 1749.5 km of survey effort in Berkeley Sound. During 13 of these survey days, there were 113 groups of sei whales sighted, totalling 210 animals (not accounting for re-sightings between days), and ranging from 1 to 18 groups and 1 to 31 animals sighted per day (electronic supplementary material, table S1). No other species of baleen whale were sighted in Berkeley Sound during the surveys. LF calls were recorded on every day of the survey effort, ranging from 10 to 929 calls per day detected in the LFDSC analysis of sei whale calls; after 22 February 2019, song was present on all but one of 12 survey days, ranging from 52 to 697 min per day logged in the manual song browse. There was a strong correlation between the daily number of whales sighted and both the number of LF calls (*r*^2^ = 0.7206) and number of minutes of song (*r*^2^ = 0.5382) per day (electronic supplementary material, figures S1 and S2).

The initial pass of the LFDCS using the North Atlantic call library resulted in over 4000 potential detections of sei whale downsweeps in the BS-Central Deployment 1 recordings. During the review of spectrograms using the LFDCS detections as a guide to locate potential vocalizations, 2394 calls were manually logged during steps 1 and 2 of the review procedure described in the methods and used to visually classify calls into discrete categories. These 2394 calls represented a subset of the many thousands of vocalizations present, in an effort to broadly review the data and identify all of the stereotyped and commonly occurring categories that were recorded on multiple days across the sample. Of this total, 510 individual vocalizations were isolated for measurement during step 3 of our review procedure based upon being good-quality signals with a high received sound level (peak power > 100 dB re 1 µPa) and a SNR > 16 dB. Calls were selected across different hours and days, to optimize the likelihood of a broad sampling of different individuals and behavioural contexts. Following that process, the resulting calls used in calculating descriptive statistics originated from 148 different hours on 46 different days between 3 January and 21 April 2019 and had a mean SNR of 33.6 dB ± 6.0 dB (range, 16.3–55.4 dB).

**Figure 2.**
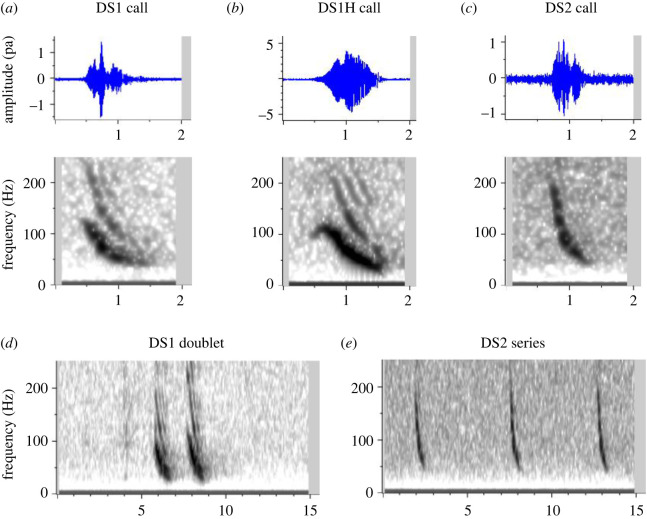
Example downsweep calls of sei whales recorded in Berkeley Sound; (*a*) a DS1 call, characterized by a high frequency less than 130 Hz and sweep from ca. 100–30 Hz; (*b*) a DS1 call with short initial upsweep, or ‘hook’ (DS1H); (*c*) a DS2 call, characterized by a high frequency > 130 Hz and sweep from ca. 160–30 Hz; (*d*) commonly occurring doublet of DS1 calls; (*e*) series of three DS2 calls. Spectrograms generated with 11 kHz sample rate, 4096pt FFT, 90% overlap, Hann window.

**Figure 3.**
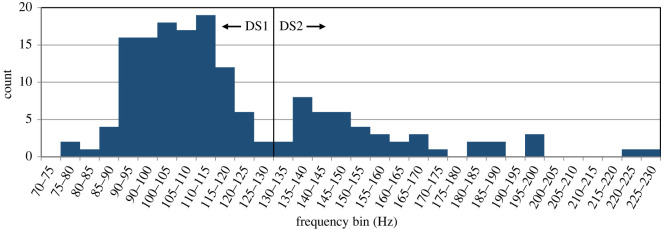
Distribution of high frequency (Hz) measurements for 157 sei whale downsweeps, indicating bimodal distribution and break between Type-1 and Type-2 Downsweeps (DS1, DS2, respectively) at 130 Hz.

**Figure 4.**
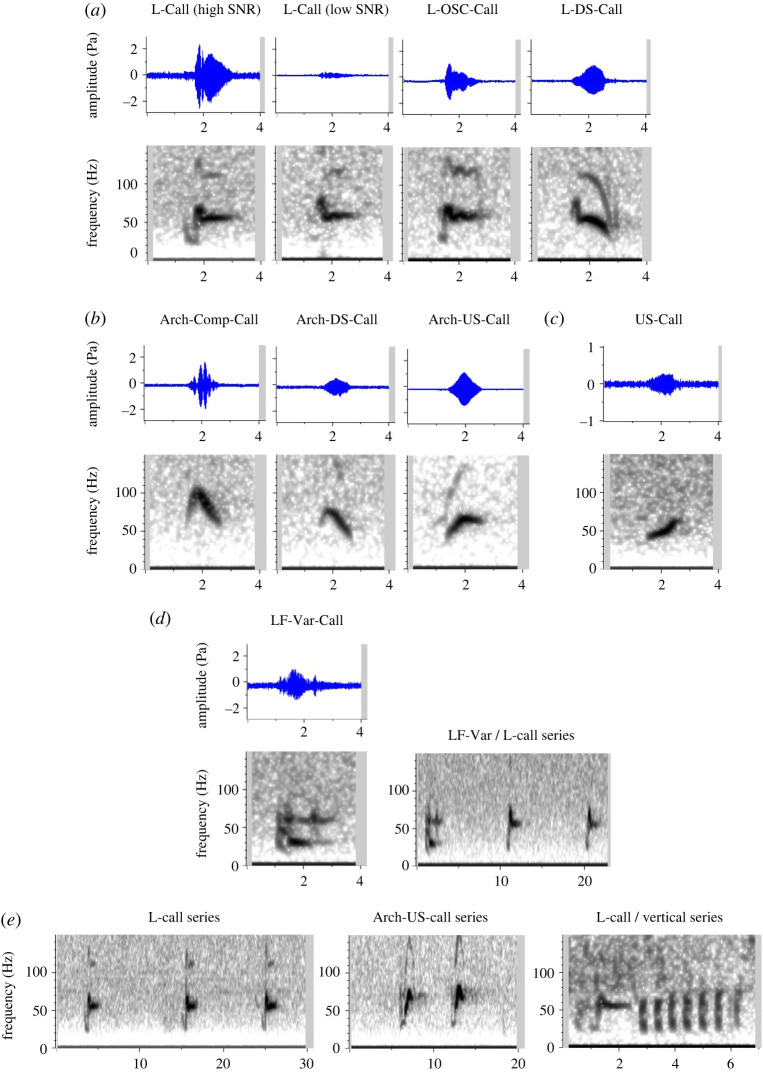
Examples of non-downsweep low-frequency vocalizations of sei whales recorded in Berkeley Sound; (*a*) examples of ‘L’-calls showing a high SNR example that illustrates the introductory ‘U’-shaped low-frequency component, a low SNR example illustrating the more commonly observed L-shaped character of the call, a variant with a frequency oscillation (L-OSC), and a variant with a terminal downsweep (L-DS); (*b*) three examples of ‘Arch’-calls, illustrating the gradations between ‘Complete Arch’ (Arch-Comp), ‘Downsweep Partial Arch’ (Arch-DS), and ‘Upsweep Partial Arch’ (Arch-US); (*c*) an example of an Upsweep (US) call; (*d*) a ‘LF Variable’-call (LF-Var), and a common series combining a LF-Var and two L-calls; (*e*) other common series of calls, including a series of three L-calls, a series of two Upsweep Partial Arch-calls, and a L-call followed by a series of grunts with a vertical spectrographic appearance. Spectrograms generated with 11 kHz sample rate, 4096pt FFT, 90% overlap, Hann window.

Five different categories of stereotyped call types were described, some with subcategories describing variation within a category. In some cases subcategories were ‘discrete’ stereotyped variations, in others they represented examples of ‘graded’ variability across the category. These included: downsweeps (at least three discrete subcategories; figures 2 and 3), L-calls (at least three discrete subcategories; figure 4*a*), arch calls (at least three graded subcategories; figure 4*b*), upsweep calls (figure 4*c*) and LF-variable calls (figure 4*d*). These categories do not necessarily represent the full range of the repertoire of calls used by sei whales in the Falklands, but rather the vast majority of commonly occurring stereotyped vocalizations that were readily identified and recognized throughout our sample.

#### 3.1.1. Downsweep calls

**Figure 5.**
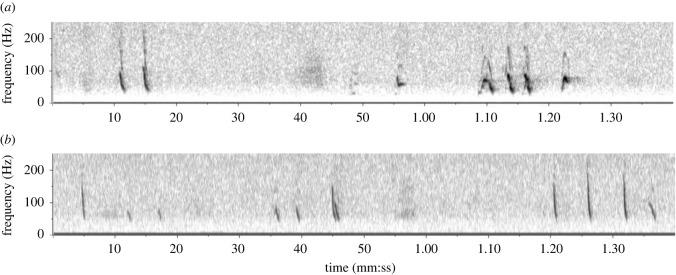
Example sequences of mixed non-song sei whale calls; (*a*) sequence showing a mix of Type-1 downsweeps, L-call, and Arch-calls; (*b*) sequence showing a mix of DS1 and DS2 downsweeps. Spectrograms generated with 11 kHz sample rate, 4096pt FFT, 90% overlap, Hann window.

A total of 157 tonal downsweeps of high SNR were chosen for quantitative measurement, spread across 57 different hours on 28 days between 14 January and 6 April 2019. At least two types of downsweep were identified: DS1 and DS2, respectively (figure 2). While there was some overlap in time-frequency characteristics, the bimodal distribution of their maximum high frequency indicated a natural break at 130 Hz (figure 3); consequently, this high-frequency threshold was adopted as a criterion to distinguish the two downsweep types, and quantitative measurements are reported on each category separately. DS1 had on average a slightly longer duration than DS2, but with broadly overlapping ranges (0.94–1.67 s versus 0.86–1.39 s; table 1). While both downsweep types ended at low frequencies of approximately 30 Hz, their high frequencies differed from average values of 105 Hz in DS1 to 158 Hz in DS2 (table 1); this resulted in a much steeper slope for DS2 (82–188 Hz s^−1^) compared with DS1 (36–95 Hz s^−1^). DS1 calls commonly exhibited a short initial upsweep or ‘hook’ (subcategory termed DS1H; figure 2*b*), as similarly described by Ou *et al*. [33] for North Atlantic sei whales. Of the 113 DS1 calls described in table 1, 49 were DS1 (without a hook) and 64 were DS1H; the two subcategories had nearly indistinguishable time-frequency characteristics, and thus quantitative measurements are reported together. None of the measured DS2 calls exhibited a hook structure. As reported for other geographical regions, DS1 at times occurred in doublets with a separation of approximately 2–4 s (figure 2*d*), but also singly or in triplets and quadruplets (e.g. [10]). DS2 were often seen in evenly spaced series with a separation greater than that between calls in a DS1 doublet (figure 2*e*), but at times also occurred in more typical doublets. The two categories were seen interspersed in the same vocalization bout on several occasions (figure 5*b*).

**Table 1.** Descriptive statistics of LF calls attributed to sei whales extracted from BS-Central during Deployment 1, between January 3 and April 6, 2019. Sample size for each category of call is indicated by: the subset of all calls that were manually logged (N of logged calls); the subset of logged calls that were quantitatively measured (*N* of measured calls); and the number of different hours and days from which measured calls were extracted (*N* of hours, days), as an indication of likely broad sampling of individuals. Spectrograms of calls are illustrated in figure 2 for downsweeps (DS1 and DS2), and figure 4 for L-calls, LDS-calls, Arch-calls, and Upsweeps. Reported values represent mean ± standard deviation (range). Signal to Noise Ratio (SNR) was measured for each measured call by comparing identical time/frequency-band selections of signal and background noise.

	call type					
	DS1	DS2	L-Call	L-DS-Call	Arch-Call	Upsweep
*N* of logged calls	342	70	1340	104	147	141
*N* of measured calls	113	44	166	46	93	48
*N* of hours, days	48, 28	23, 15	58, 26	14, 8	40, 19	22, 16
duration (sec)	1.20 ± 0.15 (0.94–1.67)	1.11 ± 0.13 (0.86–1.39)	1.9 ± 0.3 (1.33–2.7)	1.4 ± 0.1 (1.23–1.63)	1.4 ± 0.3 (0.91–2.19)	2.0 ± 0.3 (1.4–2.79)
peak freq (Hz)	57.0 ± 9.8 (38–100)	61.6 ± 13.5 (44–106)	60.0 ± 5.6 (53–73)	52.7 ± 1.9 (47–56)	66.1 ± 10.8 (41–97)	54.1 ± 6.8 (32–67)
high freq (Hz)	104.6 ± 10.7 (75–127)	158.4 ± 23.9 (131–229)	79.4 ± 3.7 (70–90)	76.6 ± 2.9 (70–82)	82.8 ± 11.1 (66–119)	70.6 ± 2.6 (66–78)
low freq (Hz)	29.9 ± 5.0 (20–46)	29.5 ± 4.8 (22–44)	22.1 ± 3.6 (16–50)	27.5 ± 4.9 (20–39)	33.0 ± 8.3 (19–65)	25.4 ± 3.2 (20–33)
slope (Hz/sec)	63.0 ± 11.6 (36–95)	117.0 ± 24.7 (82–188)	n/a	n/a	n/a	22.9 ± 3.1 (16–31)
SNR (dB)	31.9 ± 5.7 (16.7–45.2)	29.1 ± 5.7 (16.3–39.2)	35.2 ± 6.1 (19.2–53.7)	33.0 ± 5.6 (19.1–40.4)	35.2 ± 4.9 (21.9–55.4)	28.7 ± 5.8 (17.6–40.1)

In addition to downsweeps, other LF calls were commonly observed. The following distinct call types were produced with sufficient regularity to be described.

#### 3.1.2. ‘L’-call

Named due to its most typical appearance in the spectrogram of a short rapid downsweep from ca. 80 Hz to 50 Hz followed by a non-modulated tone at ca. 60 Hz with a total duration of ca. 1.5 s (figure 4*a*). High SNR examples revealed a lower amplitude initial ‘U’-shaped component between 20 Hz and 50 Hz and ca. 0.5 s in duration (figure 4*a*), resulting in a total duration of 1.9 ± 0.3 s and a mean frequency range of 22.1 ± 3.6 Hz to 79.4 ± 3.7 Hz (table 1). In a small minority of cases, the non-modulated tone displayed a frequency oscillation over approximately 10 Hz (subcategory **L-OSC** in figure 4*a*). L-calls were very common throughout the sample, highly stereotyped, and often occurred in series of three to eight calls (figure 4*e*). Another subcategory was the **‘L-DS’-call**, a variation on the L-call, with a similar short rapid downsweep from ca. 80 Hz to 55 Hz followed by a gradually curving downsweep to a low frequency of 27.5 ± 4.9 Hz, and total duration of 1.4 ± 0.1 s (figure 4*a*, table 1). L-DS-calls appeared to be relatively rare in the sample, as compared to the more common L-call. L-calls and L-DS-calls were quantitatively measured separately due to the observed discrete stereotypy of the call subcategories.

#### 3.1.3. ‘Arch’-call

Named by a characteristic inflection point at the high frequency and a reversal of contour slope from positive to negative, giving the contour a concave character. Arch-calls were among the most variable of call types (figure 4*b*), ranging from 0.91 to 2.19 s duration in the 19–119 Hz frequency band (table 1). They graded from calls that were ‘complete’ arches (Arch-Comp), to those that were ‘partial’ arches. The latter primarily comprised a downsweep partial arch (Arch-DS) or an upsweep partial arch (Arch-US) with an inflection and short hook at the start or end, respectively (figure 4*b*). Due to the graded quality of the variation within this category, Arch-calls were measured as a single group, not by subcategory.

#### 3.1.4. ‘Upsweep’-call

Tonal upsweeps (US) were observed throughout the sample, but in lower apparent occurrence than downsweeps or L-calls. They had a duration of 2.0 ± 0.3 s and occurred over frequencies sweeping from 25.4 ± 3.2 to 70.6 ± 2.6 Hz (figure 4*c*, table 1).

#### 3.1.5. ‘LF-Variable’-call

A complex low-frequency call, approximately 2 s in duration, in the 20–80 Hz band (figure 4*d*). The call displayed mild amplitude modulation at the start and ending, and a multi-harmonic tonal portion in the center with a fundamental frequency at approximately 30 Hz. The call was typically repeated in a series, or in a stereotyped pattern of a single LF-Variable call followed by two to six L-calls (figure 4*d*). Due to the complex nature of the call, the acoustic features were not measured as was done for tonal calls.

The inter-observer classification analysis was conducted to assess the robustness of call categories using the 510 high-quality calls measured in table 1, along with 50 LF-Variable calls that were not included in the quantitative dataset. The analysis revealed high agreement, with 96.4% of the 560 individual calls being categorized by the naive analyst into its originally assigned category. Four categories, DS2, L-calls, L-DS-calls and LF-Variable-calls, had 100% agreement, whereas DS1 and US calls had 96% and 98% agreement, respectively. Arch-calls had an agreement rate of 84%, with some ambiguity at the extremes of their variation between Arch-DS and DS1, and between Arch-US and US call categories.

All of these LF calls were common throughout Deployment 1 and were found in all of the months of that deployment (Dec to Apr) despite periods with few or no calls. There were often bouts of vocalizations recorded, combining the different call types without an obvious stereotyped or repetitive pattern (figure 5), as well as discrete stereotyped combinations or series of certain calls that were repeatedly observed throughout the sample (figure 2*d* and *e*, figure 4*d* and *e*).

### 3.2. Sei whale mid-frequency song

**Figure 6.**
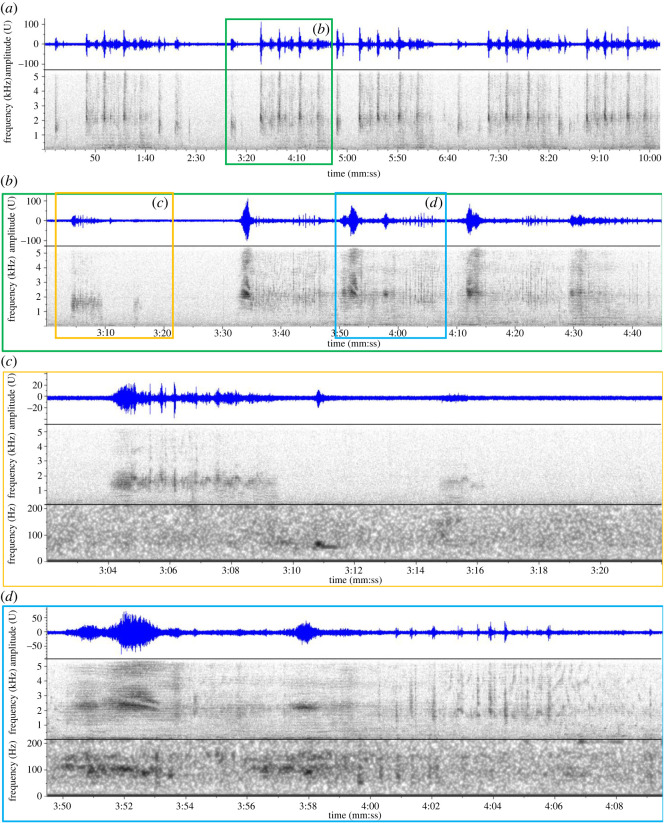
Spectrograms (lower panels) and waveforms (top panels) of sei whale mid-frequency song, with coloured boxes indicating successive details, illustrating: (*a*) 10 min sequence of five phrases (2048pt FFT, 90% overlap); (*b*) 1 min 45 s sequence of a single phrase (2048pt FFT, 90% overlap); (*c*) 20 s sequence of subphrase 1, with split spectrogram views showing full bandwidth of 0–5.5 kHz (middle panel: 512pt FFT, 90% overlap), and low frequency bandwidth of 0–200 Hz (lower panel: 2048pt FFT, 90% overlap); and (*d*) 20 s sequence of subphrase 2 motif, 2^nd^ repetition with split spectrogram views as in (*c*). All files at a sample rate of 11 kHz with spectrograms generated using a Hann window.

**Figure 7.**
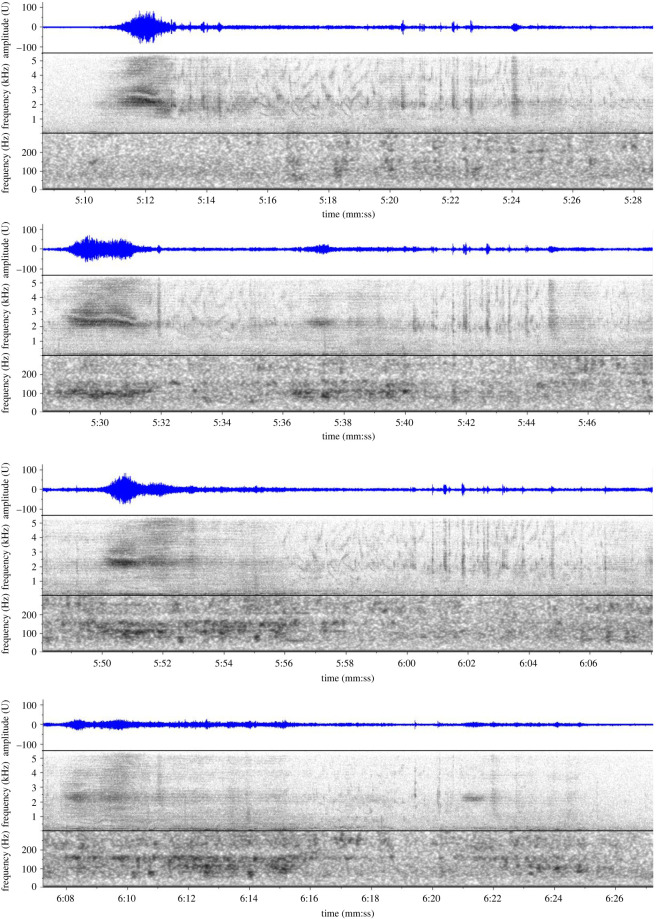
Spectrograms (lower panels) and waveforms (top panels) of sections of sei whale mid-frequency song, illustrating four consecutive repetitions of the second subphrase motif, excerpted from the third phrase in the sequence from figure 6*a*, time 5:10 to 6:26. All are rendered with split spectrogram views showing full bandwidth of 0–5.5 kHz (middle panel: 512pt FFT, 90% overlap), and low frequency bandwidth of 0–200 Hz (lower panel: 2048pt FFT, 90% overlap), at a sample rate of 11 kHz and using a Hann window.

During the manual mid-frequency scan of spectrograms from Deployment 1, complex and hierarchically organized sequences of vocalizations were discovered that were consistent with baleen whale song (figure 6). We use the terminology introduced by Payne & McVay [22] to describe the hierarchical structure of humpback whale singing behaviour, comprising units, subphrases, phrases and songs, and consider a continuous sequence of songs a bout. The individual sounds, or units, were a mix of broadband frequency sweeps in the 1.0–5.5 kHz bandwidth that had a ‘whooshing’ aural quality (figure 6*c*, 3:04 to 3:10, and figure 6*d*, 3:50 to 3:54) and sequences of noisy squeaks and creaks (figure 6*d*, 4:00 to 4:08, and figure 7, 5:13 to 5:28). The vocalizations that have been assessed to date were all arranged into a stereotyped phrase (figure 6*b*) of approximately 100 s, which can be subdivided into two distinct subphrases. The first subphrase (figure 6*c*) combines a broadband ‘whooshing’ sound (with peak energy in the 1–2 kHz frequency range), and low-frequency L-calls as part of the stereotyped pattern. The second subphrase (figure 6*d*) contains a repetitive motif that combines a broadband ‘whooshing’ sound (with peak energy in the 2.0–3.5 kHz range) and noisy squeaks and chirps and was repeated multiple times in sequence. The observed number of repetitions of the motif in the second subphrase ranged from two to five, with four repetitions appearing most common as illustrated in figure 6*b*. There were slight variations observed between successive repetitions of the second subphrase motif, including the presence of L-calls and other variable and graded LF signals below 100 Hz (figure 7). The entire phrase was repeated in a series, with short but somewhat variable timing between phrases (figure 6*a*), at times for multiple hours without pause. The application of song terminology introduced by Payne & McVay [22] to describe humpback whale song is deliberate, due to the similarities in structure. However, unlike humpback whales, which sing multiple different phrase types in a song sequence, only a single phrase type of sei whale song was identified in the Falklands' recordings. Therefore, a sei whale song comprises the repetition of a single phrase, as defined above (and thus until further analysis indicates otherwise, the term ‘phrase’ can be considered synonymous with ‘song’). Further analysis may indicate more variety and different phrase types, but currently all of the examples that were logged in BS-Central Deployment 1 conformed to the patterns described.

**Figure 8.**
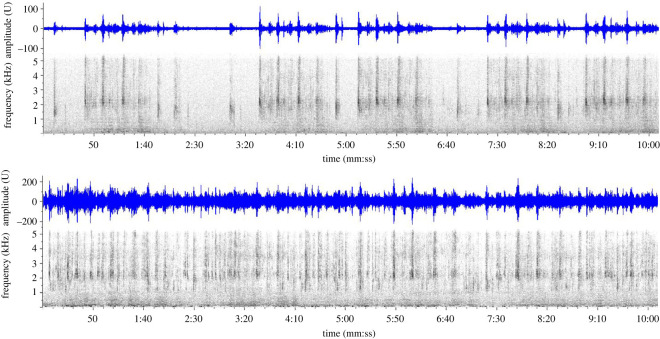
Comparison of sequences of sei whale song illustrating what is interpreted as a single singer (top panel, as is figure 6*a*), and multiple singers based upon the presence of overlapping song sequences (bottom panel). 11 kHz sample rate, 2048pt FFT, 90% overlap, Hann window.

During the systematic review of BS-Central Deployment 1, sei whale songs were recorded during a total of 408 one-hour periods on 56 days, in 267 logged bouts totalling 191.4 h (with a ‘bout’ defined as a continuous sequence of songs with no gaps greater than 10 min, roughly the duration of five consecutive songs in an uninterrupted sequence). Bout duration was measured on 35 bouts of the highest quality (scoring 3 on our 1–3 qualitative scale) in order to limit the assessment to a subsample of the closest and clearest singing whales. Mean bout duration was 90.3 m (s.d. 51.7 m) with a range of 17.1 to 211.5 m, when considering only these 35 high-quality bouts. Whereas bouts of lower quality had shorter mean durations (45.9 m and 17.5 m, for quality 2 and 1, respectively), the longest recorded bout was a quality 2 bout of 245.9 m. In most cases, a bout of singing faded into the recording at the start and faded out at the end, indicating that the singing whale(s) moved within and then out of detection range of the recorder and singing was likely occurring prior to and after the recorded bout period. Therefore, bout durations are indicative of a minimum singing duration. The complexity of the sounds that comprised the songs made it difficult to determine how many whales were singing when there were multiple singers; however, it was fairly evident when there was a single ‘solo’ singer versus multiple singers in the recording (figure 8). Again considering only the 35 highest quality bouts, only 8 bouts were scored as a solo singer, whereas 27 bouts (77%) were scored as having multiple singers; of those, 23 appeared to have at least 2 singers, and 4 likely had 3 or more singers. Therefore choruses of whales appeared to be common, and the reported bout durations may not represent a single whale.

**Figure 9.**
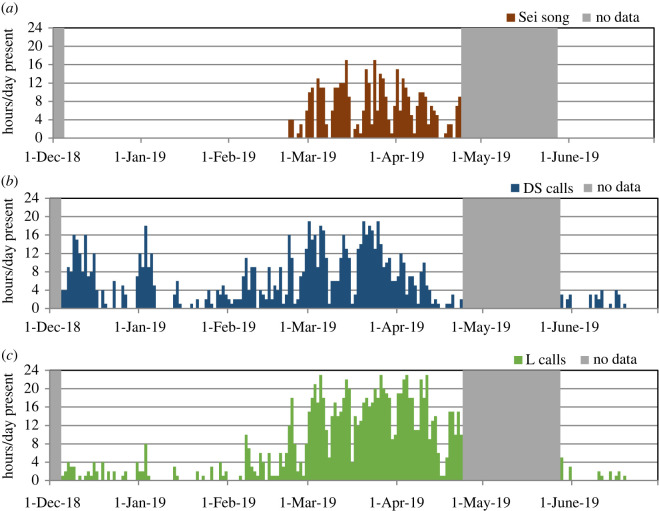
Hourly presence of sei whale song and calls at BS-Central from 5 December 2018 to 30 June 2019: (*a*) mid-frequency song; (*b*) downsweep (DS) calls; and (*c*) L-calls.

An assessment of hourly presence of song at BS-Central (figure 9*a*) revealed that sei whale song was not recorded during December 2018, January 2019 or early February 2019, and the first occurrence was detected on 22 February 2019. It was anecdotally noted that singing in Berkeley Sound commenced at least one week earlier, as it was recorded on the BS-Outer recorder as early as 14 February 2019; however, a systematic review of the BS-Outer recorder for occurrence of song has not yet been undertaken. Song was recorded at BS-Central on 56 days between 22 February and 23 April and during an average of 7.2 h per day (range of 1 to 17 h). The commencement of singing in mid to late February is in contrast to the detection of non-song vocalizations (downsweeps, and L-calls not incorporated into song sequences), which were detected throughout December and January (figure 9*b,c*). Song was recorded extensively during March and April 2019 and was ongoing when the recorder was retrieved on 23 April 2019. Concurrent with the commencement of, and increase in, singing, there was a dramatic increase in the detection of L-calls over the same period (figure 9*c*), apparently due to the incorporation of the L-call into song patterns. After the month-long gap in recording during most of May, songs were not detected again at BS-Central during the start of Deployment 2 (reviewed through 30 June 2019). The occurrence of downsweeps and L-calls was also greatly reduced during June, but those calls were not entirely absent until after mid-June (figure 9). It is recognized that whales move away from the Falklands around late May/early June, as documented previously and during this study in both the visual surveys and acoustic monitoring [3,21,32].

**Figure 10.**
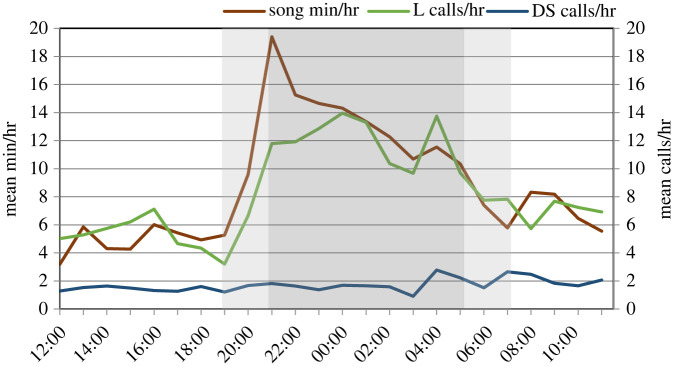
Diel variation in occurrence of song (in mean min h^−1^), and in downsweeps (DS) and L-calls (in calls/h), during the predominant period of singing from 1 March to 23 April 2019. Light grey shading indicates astronomical twilight and dark grey shading indicates night-time hours (between sunset and sunrise), with divisions representing the average over the time period.

Diel variation (assessed during the predominant period of singing from 1 March to 23 April 2019) indicated that sei whales sang more at night than during the day, with a dramatic increase in singing activity at the start of astronomical evening twilight, and a peak at sunset (figure 10). Singing activity remained high relative to daytime, while gradually declining through the night, and then dropped during morning twilight to a low at sunrise, remaining relatively low through the daylight hours with a minimum at noon. The occurrence of L-calls closely tracked the same diel pattern as singing activity, whereas downsweeps displayed a comparatively lower occurrence rate but remained consistent throughout daylight, twilight and night-time hours (figure 9).

## 4. Discussion

### 4.1. Attribution of vocalizations to sei whales

We have here described the vocal repertoire of sei whales in the Falkland Islands and the western South Atlantic for the first time. Given the nature and extent of our acoustic dataset relative to previous studies, we have also been able to describe the vocal behaviour of the species in greater detail than other studies globally, adding several previously undocumented sei whale vocalization types. The success of any PAM study using remote recorders is dependent on several factors, including the ability to confidently assign vocalizations to a particular species, the density of whales in the study area and the consequent probability of their vocalizing within detection range of a recorder, and the presence/absence of other species that may produce confounding similar vocalizations. In Berkeley Sound, species attribution of vocalizations was facilitated by descriptions of sei whales calls from other geographical areas, and previous and concurrent boat surveys in Berkeley Sound that provided information on the relative occurrence of baleen whale species at the site. Specifically, five seasons of cetacean survey work have evidenced the routine and extensive use of the site by sei whales (confirmed by genetics and photo-identification), including over the months of acoustic monitoring analysed here [3,21]. The documented predominance of sei whales at the site, and the relative scarcity of other baleen whale species at that time of year [3,21], allowed previously undocumented vocalizations that occurred in bouts among known sei whale vocalizations to be positively attributed to that species. With over 200 sei whales sighted during nearly 2000 km of survey effort and no other baleen whales documented during the recording period, along with the high rate of occurrence of all of the described vocalizations, we conclude that the species attribution to sei whales is unequivocal.

It is worth commenting here on relevant results from an unpublished report to a petroleum industry company [34]. During remote acoustic monitoring carried out 230 km north of the Falklands in 400 m depth during 2013, sounds were recorded that were identical to segments of the sei whale song reported here, primarily between late February and early April ([34], p. 70). Without the availability of concurrent visual data, those authors attributed the sounds as aberrant calls made by leopard seals (*Hydrurga leptonyx*) based upon a superficial similarity to vocalizations made by immature males of that species [35]. Although leopard seals have historically been reported in the Falklands [36,37], they have only been observed three times during boat-based surveys in the Falklands in 2017–2022, and all during the winter (in June and July, Falklands Conservation, unpublished data). Moreover, none have ever been observed during our five years of whale surveys in Berkeley Sound, where our acoustic deployments occurred. While we cannot rule out an occurrence of leopard seals in offshore Falklands’ waters, given the seasonality and characteristics of the sounds reported in Hipsey *et al*. [34], we consider that their spectrograms illustrate portions of sei whale song rather than aberrant leopard seal calls.

Falklands’ waters are recognized as supporting an internationally important concentration of sei whales, with densities recorded around the Islands exceeding those recorded in other geographical regions by at least an order of magnitude [6], and therefore yielding high numbers of vocalizations for analysis. Furthermore, the specific qualities of Berkeley Sound, a small (approx. 20 × 6 km) and semi-enclosed embayment with rich natural resources results in whales foraging continuously on lobster krill for periods of days to weeks ([3]; Falklands Conservation unpublished satellite tracking data) within a relatively confined area, which optimizes the quality (SNR) and abundance of the vocalizations available for analysis. These factors in combination have facilitated high confidence in species attribution. For example, species attribution would have been considerably more challenging in the oceanic areas often inhabited by sei whales worldwide, where acoustic monitoring is often selected instead of boat surveys due to logistics and cost considerations, but then lacks visual confirmation of species identification. Similarly, species attribution would have been more difficult in areas where other baleen whale species co-occur temporally with sei whales, for example off Massachusetts and the Gulf of Maine [16].

### 4.2. Low-frequency calls

Several discrete LF-calls were described and confidently attributed to sei whales, including some that were similar to calls previously reported from other regions, and others that were novel. The DS1 downsweeps that we described correlate broadly with an LF downsweep that has been widely documented in both the Southern and Northern Hemispheres [9–13]. With a frequency range of 127 to 20 Hz, DS1 are most similar to the 41 sei whale downsweeps reported by Español-Jiménez *et al*. [12] in the eastern South Pacific (off Chile) which had a reported frequency range of 129–30 Hz. The downsweeps from these two Southern Hemisphere locations were slightly higher than those reported for Northern Hemisphere sei whales, which generally sweep from approximately 100–30 Hz [9,10,13]. By contrast to DS1, the DS2 downsweep that we reported is novel and had a much higher maximum frequency and steeper slope then has been previously reported for sei whale LF downsweeps. The maximum frequency of DS2 calls in the Falklands ranged from 131 to 229 Hz, whereas other studies do not report any LF downsweeps with a maximum frequency in excess of 130 Hz [9,11,12]; although it is noted that McDonald *et al.* [19] report an MF downsweep from ca. 600–300 Hz off the Antarctic peninsula that has not been recorded elsewhere. Therefore, the acoustic features of DS2 do not overlap with previously reported LF downsweeps and can be considered a new call-type for sei whales. In addition, the L-calls, and their associated variants L-OSC and L-DS-calls, do not appear to have been described in the literature, and thus are completely novel. The correlation of the occurrence of L-calls with the documented song display in the latter half of the recording period (see below) suggests that these calls may be associated with male breeding behaviour. Unlike the song display, L-calls were also recorded in relatively lower numbers during the early half of the recording season, when song was not recorded, and therefore they may serve multiple behavioural functions. Arch calls in the Falklands were similar to the upsweep-downsweep call described previously by Calderan *et al.* [11] from the Southern Ocean, but with a broader frequency range of 19–119 Hz compared to their 49–79 Hz. This discrepancy may be due to the small sample size of Calderan *et al*. (*n* = 4) compared to the 93 high SNR examples that were measured in the Falklands. The frequency range of upsweeps in the Falklands (20–78 Hz) overlapped broadly in frequency with those in the Southern Ocean (34–76 Hz [11]), but were much longer in duration (1.4–2.8 s compared to 0.4–1.7 s, respectively) and thus presented a shallower slope than those recorded in the Southern Ocean.

It is important to recognize that sample sizes in most previous studies that report on the sei whale vocal repertoire were small relative to our available sample of thousands of calls, due to the low density of sei whales and/or the opportunistic nature of the recording events in other studies (e.g. [19]: 50 tonal and 18 frequency swept calls during a single 1-hour event; [9]: 107 downsweeps during a single 2-h event; [11]: four downsweeps, 30 upsweeps, 4 upsweep-downsweeps during a single 1.5-h event; [14]: 61 downsweeps during a single 2.5-h event; [12]: 41 downsweeps during three 30-min events), and in many cases, the apparent SNR illustrated in published spectrograms is very low (e.g. [19]). This likely limited both the representativeness of the sei whale vocal repertoire (due to minimal recording opportunities and consequent small sample sizes) and the spectrographic representation and accurate measurement of acoustic features of calls (due to relatively low SNR), although some of the observed differences may also be attributed to geographical and seasonal variation. This again emphasizes the uniqueness of the Berkeley Sound field site, as a small isolated body of water with a seasonally high density of sei whales. The consequent availability of large sample sizes of high SNR calls in the Falklands dataset has facilitated the most comprehensive description of sei whale call types that has been published to date. Given the worldwide distribution of sei whales, it is expected that this result will inform numerous acoustic monitoring programmes globally in identifying the species, which in turn will provide a greater understanding of its distribution, ecology and behaviour.

### 4.3. Mid-frequency song

Possibly the most consequential finding of this work on a global scale is the discovery of mid-frequency song in sei whales. Song has been documented among most of the Balaenopteridae, and is suspected to be a common male reproductive display. Singing behaviour has been best described for humpback whales (reviewed in [38]), but most species of *Balaenoptera* produce rhythmically repetitive vocalizations that have formally been recognized as songs, including blue whales [25], fin whales [23,24], Antarctic minke whales (*B. bonaerensis*, [39]), common and dwarf minke whales (*B. acutorostrata*, [40–42]), and Omura's whales (*B. omurai*, [26,27]). Bryde's whales (*B. edeni*) and Rice's whale (*B. ricei*) have also been reported to make stereotyped ‘calls’ [sic] that have rhythmic repetition rates [43–46]; the descriptions of these vocalizations are consistent with a reproductive song display, although they are not formally referred to as song in their respective literature. Therefore, it appears that singing and use of stereotyped rhythmic song is an ancestral trait common among all members of Balaenopteridae, and we should expect to find a song display in the vocal repertoire of sei whales. However, unambiguous stereotyped song has not been conclusively reported previously for sei whales. The series of different downsweep types reported by Tremblay *et al*. [13] for North Atlantic sei whales appear to be organized in song-like patterns, and the stereotypy of the sequences and consistency of song structure warrants further evaluation and description. In our assessment of sei whale vocalizations in the Falklands, sequences of LF downsweeps resembling those reported by Tremblay *et al*. [13] were never observed. Rather, the Falklands' songs occurred in a different frequency band (up to 4000 Hz) and were composed of different and highly varied vocalization types (complex combinations of broadband mid-frequency sweeps, mid-frequency chirps and creeks and low-frequency vocalizations). Moreover, they presented a highly stereotyped and hierarchical structure consistent with complex balaenopterid song, and similar to the phrase structure of humpback whales [22,47].

Sei whale songs were absent in Berkeley Sound early in the recording period from December to February, but became common in early March, and continued through the remaining period of sei whale presence. Such distinct seasonality is congruent with a reproductive song display. Furthermore, diel variation of singing peaking during night is similar to diel patterns in singing activity documented in other balaenopterids, such as humpback whales [48–50] and blue whales [51,52]. Further analysis should seek to confirm the seasonality, to assess whether song consistently commences across the Falkland Islands during the austral autumn (i.e. inter-annually and at other sites) and whether it continues into the winter elsewhere in the migratory range. Within a comparative framework, the observed seasonal pattern of occurrence is similar to what has been well described for the seasonality of humpback whale vocal behaviour in high latitude feeding areas, where singing is rarely recorded during the summer months, but commences during the autumn before males migrate to low latitude breeding areas [53–55]. This overlap of singing behaviour and feeding habitat has been linked to seasonal hormonal/physiological changes that occur in autumn on high latitudes, and likely drive the onset of breeding behaviour and seasonal ontogeny of singing [38,56]. The commencement of singing by sei whales in the Falkland Islands appears to follow a similar pattern, and it is proposed that singing may continue through the winter during the migration of whales to their lower latitude wintering destinations (e.g. offshore Arraial do Cabo, Brazil [5]).

This novel finding has several important implications that are broad reaching. The existence of MF song in sei whales is a substantial contribution to the global understanding of sei whale behaviour and will facilitate researchers in other geographical regions to appropriately explore existing and future datasets, and potentially identify the presence of sei whale song. Since singing in Balaenopterids is considered a breeding display produced by males [24,47,57,58], consequently, this is also likely true for sei whales. Therefore, documenting singing is useful not only as an indicator of presence of sei whales in a region, but also as an indication of a reproductive display that is likely integral to sei whale life history. Moreover, the occurrence of sensitive behaviours such as breeding activity has management implications for any habitat and region in which they are found.

Given the relatively high rate of occurrence of song recorded throughout March and April in Berkeley Sound, it is somewhat puzzling that this specific song display has not been reported previously in other geographical regions. This is possibly due to a combination of study-specific factors, including lack of monitoring at appropriate locations and latitudes and times of year, along with an analytical focus on low frequencies given that most previously described sei whale vocalizations are below 500 Hz. Many datasets are in fact limited by low sample rates that would not record the predominant frequency band of this song (e.g. from the MARUs used in [16], and some recorders in [15], recording at 2 kHz sample rate). In other studies that record at an adequate bandwidth, it seems possible that the song display was overlooked due to an analytical focus on the automated detection of LF sounds, specifically LF downsweeps (e.g. [15,20], in which only a subsample of data was manually evaluated for validation of LF detectors, and spectrogram parameters are not reported). Furthermore, the acoustic characteristics of the sei whale song described here likely limits its propagation over long distances, so a recorder would need to be in relatively close proximity to a singing whale for accurate documentation. Moreover, we recorded these songs among dense aggregations of sei whales, in which complex social interactions are likely to be a prominent behaviour; it is possible that the same social context was not common in other studies, because they were not working with aggregations of sei whales at sufficiently close proximity. This again emphasizes the uniqueness of the Berkeley Sound field site, with the opportunity to record many individuals in close proximity for extended periods.

Conversely, it is conceivable that the mid-frequency song described here does not exist in the North Atlantic (or Northern Hemisphere in general), and that the North Atlantic population of sei whales may use only a low-frequency song as described by Tremblay *et al*. [13]. This seems at least possible given that extensive datasets have been collected at adequate sample rates, during autumn and winter months on the Scotian Shelf [20] and off the Azores [15], but no similar MF song display has been reported. However, we note that this would be phylogenetically and evolutionarily incongruent, since the fundamental characteristics of song structure (in terms of frequency band, unit and phrase structure and timing/rhythm of song components) tend to be conserved among populations of the same species, such that their songs are generally recognizable as that species despite geographical variation among the populations. This consistency in song structure has been well demonstrated for humpback whales [38,59], blue whales [25], minke whales [39] and Omura's whales [27,60]. The MF song that we describe is so acoustically divergent from the LF vocalizations reported in other populations, that it is difficult to imagine a selective scenario in which populations of the same species could evolve such divergent reproductive displays. Furthermore, Thompson *et al*. [17] and Knowlton *et al.* [18] cursorily reported sound types from North Atlantic sei whales that appear to resemble the song units we described, with a similar frequency range and aural quality. Knowlton *et al.* [18] recorded these sounds on the Scotian shelf during the boreal autumn months, so suggesting a similar seasonality for a high-latitude feeding area. Future research efforts should search for this song display in existing or future acoustic datasets, for any region where sei whales have been documented in modern acoustic or visual surveys. In the western South Atlantic, effort should particularly focus on Arraial do Cabo and Costinha, Brazil, regions thought to be one breeding destination during the austral winter for the population that summers in the Falklands [5].

It is noteworthy that the acoustic characteristics of the sei whale song we have described diverge substantively from that of the species' congeners. Song in most *Balaenoptera* spp. (apart from the minke whale species) tends to be low frequency with infrasonic components, produced at powerful source levels, and without complex acoustic structure, and thus appears to be selected for optimizing long distance propagation and communicating among widely dispersed receivers [61]. Conversely, sei whale song in the Falklands is composed of broadband units with predominant energy in the mid-frequency 1–5 kHz band, combined with a complex arrangement of short chirping and sweeping units in the same band, and diffuse energy in the lower frequencies. The phrases of the song have complex hierarchal structure (figure 6) with subtle but consistent variation between repeated subphrase components (figure 7). Furthermore, the SNR of recorded songs did not approach the more powerful tonal LF-calls recorded in the same environment, and thus the source level does not appear to be particularly high, as would be expected for signals in this frequency band and of this structure. Therefore, we suggest that this display was not selected for long-distance propagation, as the songs of blue and fin whales for example, and it is likely a predominantly short-distance display. This has implications for the breeding behaviour of sei whales, of which virtually nothing is known. While this sei whale song is an outlier among its congeners, some of these spectral characteristics are generally shared with humpback whale song, which has complex phrasing and combinations of low and mid-frequency components. Murray *et al*. [62] proposed that different components of humpback whale song are selected for different functions, combining simple long-distance communication LF elements, with more complex MF structures that could only be perceived and evaluated at close distances, and thus the song likely serves as a multi-message display. Sei whale song similarly contains some simple LF components (L-calls) that may communicate the presence of singing whales at greater distance, as proposed for the LF components of humpback song. However, the predominant information in the display (i.e. for a female evaluating a singing male, or for competing males assessing each other) appears to be in the complex MF structures that can only be assessed at close distance. Given these characteristics, we might predict that once winter breeding aggregations of sei whales are identified and studied, these displays are used among relatively closely spaced individuals, possibly in shallower shelf habitat, as opposed to deep water abyssal plain habitat.

Singing also has implications for the detectability of vocalizing sei whales which may be higher for males engaged in prolonged bouts of singing, depending on the vocalization type that is used for detection. Care should be taken in interpretation of the temporal distributions of specific vocalization types. The close correlation of seasonality and diel patterns of L-call occurrence with singing behaviour is indicative of the L-call being a component in the song, and may therefore potentially be male-specific. Moreover, the dramatic increase in the presence of L-calls starting in late February appears to be at least in part due to a change in vocal behaviour (commencement of singing), and not solely due to an increase in number of animals as indicated by the visual surveys during this period [21]. Conversely, downsweeps were recorded throughout the entire period of sei whale detections at a relatively more consistent rate then L-calls, indicating that they may be produced by both sexes (as shown for blue whales [58]).

### 4.4. Conservation considerations

Given the occurrence of significant numbers of sei whales, and important feeding and reproductive behaviours documented here and in previous studies (e.g. [3,21]), the potential for acoustic masking and disturbance in Berkeley Sound is possibly of high conservation importance. Berkeley Sound is one of the busiest areas of the Falklands for marine traffic and is used extensively during the fishing seasons for anchoring, transhipments and bunkering. Anthropogenic ocean noise is increasingly considered to represent a stressor for marine mammal populations globally [63,64]. The introduction of noise from large vessels has the potential to impact whale populations both directly, through disturbance that can cause displacement from important areas or cessation of important behaviours (e.g. feeding or breeding), or indirectly, by causing changes in prey, by masking important vocalizations such as breeding displays [65,66], or by increasing stress and raising consequent levels of stress hormones that can add to cumulative impacts on individuals’ health [67]. Noise from large ships was a major component of the Berkeley Sound acoustic soundscape, with ships raising the ambient noise level by up to 50 dB RMS re: 1 µPa documented in the 50–200 Hz frequency band [32]. Studies should be undertaken to assess the potential risks and impacts of commonly introduced noise and make subsequent recommendations for managing anthropogenic noise to limit disturbance to local whale populations.

## Data Availability

Data are provided as electronic supplementary material, in the form of acoustic .wav files of all exemplar sounds illustrated by the spectrograms in the manuscript figures, and an excel file with the databases of measurements on calls and song data [68].
